# Persistent thinness and anorexia nervosa differ on a genomic level

**DOI:** 10.1038/s41431-023-01431-8

**Published:** 2023-07-20

**Authors:** Christopher Hübel, Mohamed Abdulkadir, Moritz Herle, Alish B. Palmos, Ruth J. F. Loos, Gerome Breen, Nadia Micali, Cynthia M. Bulik

**Affiliations:** 1 Social, Genetic & Developmental Psychiatry Centre, Institute of Psychiatry, Psychology & Neuroscience, King’s College London, London, UK; 2 National Institute for Health Research (NIHR) Maudsley Biomedical Research Centre at South London and Maudsley NHS Foundation Trust, London, UK; 3 National Centre for Register-based Research, Aarhus Business and Social Sciences, Aarhus University, Aarhus, Denmark; 4 Department of Medical Epidemiology and Biostatistics, Karolinska Institutet, Stockholm, Sweden; 5 Department of Pediatric Neurology, Charité – Universitätsmedizin Berlin, Berlin, Germany; 6 Department of Psychiatry, Faculty of Medicine, University of Geneva, Geneva, Switzerland; 7 Department of Biostatistics & Health Informatics, Institute of Psychiatry, Psychology & Neuroscience, King’s College London, London, UK; 8 Charles Bronfman Institute for Personalized Medicine, Icahn School of Medicine at Mount Sinai, New York, New York, USA; 9 Novo Nordisk Foundation Center for Basic Metabolic Research, Faculty of Health and Medical Science, University of Copenhagen, Copenhagen, Denmark; 10 Great Ormond Street Institute of Child Health, University College London London, UK; 11 Mental Health Services in the Capital Region of Denmark, Eating Disorders Research Unit, Psychiatric Centre Ballerup, Ballerup, Denmark; 12 Department of Psychiatry, University of North Carolina at Chapel Hill, Chapel Hill, NC USA; 13 Department of Nutrition, University of North Carolina at Chapel Hill, Chapel Hill, NC USA

**Keywords:** Development, Psychology

## Abstract

Thinness and anorexia nervosa are both characterised by persistent low weight. Individuals with anorexia nervosa concurrently report distorted perceptions of their body and engage in weight-loss behaviours, whereas individuals with thinness often wish to gain weight. Both conditions are heritable and share genomics with BMI, but are not genetically correlated with each other. Based on their pattern of genetic associations with other traits, we explored differences between thinness and anorexia nervosa on a genomic level. In Part 1, using publicly available data, we compared genetic correlations of persistent thinness/anorexia nervosa with eleven psychiatric disorders. In Part 2, we identified individuals with adolescent persistent thinness in the Avon Longitudinal Study of Parents and Children (ALSPAC) by latent class growth analysis of measured BMI from 10 to 24 years (*n* = 6594) and evaluated associations with psychiatric and anthropometric polygenic scores. In Part 1, in contrast to the positive genetic correlations of anorexia nervosa with various psychiatric disorders, persistent thinness showed negative genetic correlations with attention deficit hyperactivity disorder (*r*_gAN_ = 0.08 vs. *r*_gPT_ = −0.30), alcohol dependence (*r*_gAN_ = 0.07 vs. *r*_gPT_ = −0.44), major depressive disorder (r_g__AN_ = 0.27 vs. r_g__PT_ = −0.18) and post-traumatic stress disorder (r_gAN_ = 0.26 vs. r_gPT_ = −0.20). In Part 2, individuals with adolescent persistent thinness in the ALSPAC had lower borderline personality disorder polygenic scores (OR = 0.77; *Q* = 0.01). Overall, results suggest that genetic variants associated with thinness are negatively associated with psychiatric disorders and therefore thinness may be differentiable from anorexia nervosa on a genomic level.

## Introduction

Individuals with thinness (referred to by several terms—See Box 1) have persistently low body mass indices (i.e., BMIs < 18.5 kg/m^2^) [1] as defined by the World Health Organization (WHO) [2] and show no symptoms of disordered eating [3]. Persistence of low BMI is an important component of the phenotype and requires longitudinal assessment. In some cases, the phenotypic definition is augmented by a family history of thinness as an additional criterion [4]. However, definitions of thinness vary considerably across studies [5]. Although the actual prevalence of thinness is unknown [1], published reports suggest that it is relatively uncommon, estimated to be less than 0.4% for men and less than 2.7% for women (underweight from all causes) [6]. Attempts to gain weight in individuals with thinness can be complicated as individuals may resist dietary supplementation (e.g., with high-fat foods), as seen in a small controlled study [7].

Thinness shares only a few features with the restricting subtype of anorexia nervosa. A BMI < 18.5 kg/m^2^ is the primary shared symptom, as individuals with anorexia nervosa display restrictive eating behaviours, body dissatisfaction, and distorted perceptions of their own shape and weight [8]. Although individuals with thinness may also display body dissatisfaction, generally, they accurately perceive their thinness and typically want to gain, rather than lose, weight. However, the risk for potential misdiagnosis exists [1]. Women with thinness have lower levels of general psychopathology reporting lower body dissatisfaction and drive for thinness [3], and fewer depressive and anxiety symptoms [9] than controls. However, the frequency of psychiatric disorders (e.g., alcohol use disorder, generalised anxiety disorder, major depressive disorder, panic disorder, and phobias) has been reported to not differ between women with and without thinness [3]. In men, however, constitutional thinness has been reported to be associated with an adverse mental health profile, potentially due to the discrepancy between their body size and the masculine muscular ideal [10]. In this investigation, we are using three different definitions of thinness to differentiate among phenotypes, for details, please, see Box 1.

Human body weight regulation is under considerable genetic control [12] and the first genome-wide association study (GWAS) of persistent thinness estimated its single nucleotide polymorphism-based heritability (SNP-based *h*^2^) at 28% [11], indicating that a substantial proportion of the trait variance is associated with common genomic variants in a sample of individuals with thinness ascertained by health professionals (for details, see Methods). This persistent thinness phenotype showed a small and statistically non-significant genetic correlation with anorexia nervosa (*r*_g_ = 0.13; 95% CI –0.02, 0.28; *p* = 0.09) [11]. Riveros-McKay et al. drew a replication cohort from the UK Biobank: 3532 individuals with a BMI ≤ 19 kg/m^2^, excluding individuals with health conditions that could account for their thinness. This replication thinness phenotype was positively genetically correlated with anorexia nervosa (r_g_ = 0.49, 95% CI 0.22, 0.76; *p* = 0.0003); however, their original persistent thinness phenotype and their replication phenotype only showed a genetic correlation of r_g_ =  0.62 (95% CI 0.20, 1.00; *p* = 0.004), indicating that the replication phenotype only partially overlaps with the original thinness phenotype.

Anorexia nervosa shows genetic correlations with other psychiatric disorders, including obsessive-compulsive disorder, major depressive disorder, anxiety disorders, and schizophrenia [13]. Whether thinness shows a similar pattern of genetic associations as seen in anorexia nervosa is unknown. Therefore, the rationale for this current paper is as follows. We aim to replicate the finding that anorexia nervosa and persistent thinness are not significantly genetically correlated in a larger sample than previously studied. In addition, our analyses go beyond what is currently available in the literature, estimating new, previously unreported genetic associations between persistent thinness and other psychiatric traits, and contrasting these with the associations seen in anorexia nervosa. Finally, we use polygenic scores of psychiatric and anthropometric traits to predict persistent thinness in a separate longitudinal population-based cohort spanning childhood to young adulthood, going beyond previous research which focussed exclusively on adulthood.

Our overall aim was to examine differences between thinness and anorexia nervosa on a genomic level. For this, we used two genetically-informed approaches and formulated the following hypotheses:

1. In Part 1, using published GWAS results, we hypothesised that persistent thinness and anorexia nervosa would not be genetically correlated and that persistent thinness [11] would not share the same pattern of genetic correlations with psychiatric disorders seen in anorexia nervosa [13].
2. In Part 2, using polygenic scores in the Avon Longitudinal Study of Parents and Children (ALSPAC), we hypothesised that an adolescent persistent thinness phenotype (derived for the purpose of this study by us; Box 1) would be associated with polygenic scores for persistent thinness and obesity, but not with psychiatric disorders.

Box 1 Definitions of thinness in this manuscript

**Table:** 

Term	Definition
1) Thinness	Umbrella term for the phenotype: persistent low body mass indices (i.e., BMIs < 18.5 kg/m^2^), no disordered eating, average physical activity, no intake of medication or drugs, no somatic illness or psychiatric disorders that better explain the low weight
2) Persistent thinness [11]	Definition used in the genome-wide association study by Riveros-McKay et al. (2019): a BMI ≤ 18 kg/m^2^; no chronic renal, liver, gastrointestinal, metabolic disease, psychiatric disorder, or eating disorder. British individuals aged 18–65 years.
3) Adolescent persistent thinness	We derived this thinness phenotype in the Avon Longitudinal Study of Parents and Children (ALSPAC) from repeated measures of BMI (British individuals aged 10–24 years) using Latent Class Growth Analysis, excluding those with disordered eating (e.g., fasting) and eating disorders.

## Methods

### Part 1

To estimate the genetic overlap between persistent thinness/anorexia nervosa and psychiatric disorders, we obtained GWAS summary statistics from published studies: ADHD [14], anxiety disorders [15], autism spectrum disorder [16], bipolar disorder [17], borderline personality disorder [18], major depressive disorder [19, 20], obsessive-compulsive disorder [21], posttraumatic stress disorder [22], alcohol dependence [23], and schizophrenia [24] (for detailed description, see Supplementary Table S1). We included two genetic correlations from Watson et al. 2019 (i.e., anxiety disorder & obsessive-compulsive disorder) as no new GWAS on those disorders had been published since the publication of Watson et al.

### Anorexia nervosa genome-wide association study

The published GWAS of anorexia nervosa contained 16,992 cases and 55,525 controls, including 72,358 females (16,531 of whom are cases) and 24,454 males (460 of whom are cases) [13]. It was led by the Anorexia Nervosa Genetics Initiative [25] and the Eating Disorders Working Group of the Psychiatric Genomics Consortium (PGC-ED; www.med.unc.edu/pgc) meta-analysing 33 cohorts from 17 countries. The GWAS was augmented with samples from the Genetic Consortium for Anorexia Nervosa (GCAN), the Wellcome Trust Case Control Consortium 3 [WTCCC-3; [26], and the UK Biobank [27]. Case definitions established a lifetime diagnosis of AN via hospital or register records, structured clinical interviews, or online questionnaires based on standardised criteria—DSM-III-R, DSM-IV, ICD-8, ICD-9, or ICD-10—, whereas in the UK Biobank cases self-reported a diagnosis of AN [28]. The GWAS showed a SNP-based heritability of 17%.

### Persistent thinness genome-wide association study

The published GWAS of persistent thinness contained 1471 individuals with thinness and 10,433 controls, including 7162 females (1325 of whom are cases) and 4893 males (297 of whom are cases) [11]. Persistent thinness was defined as ≤ 18 kg/m^2^ and based on detailed medical and medication history from general practitioner (GP) records in British individuals aged 18–65 years. The case notes of each potential participant were reviewed by the GP or a senior nurse with clinical knowledge of the participant. Individuals with chronic renal, liver, gastrointestinal, metabolic diseases, psychiatric disorders, or eating disorders, and high physical activity (i.e., > 3 times per week) were excluded. Additionally, individuals were screened for eating disorders using the SCOFF questionnaire [29] and needed to endorse the question that they always had been thin to establish persistence. This stringent exclusion of individuals with psychiatric disorders is an essential part of the thinness definition when conducting a GWAS; however, it represents a limitation when calculating genetic correlations with psychiatric disorders. The GWAS showed a SNP-based heritability of 28%.

### Linkage disequilibrium score regression to calculate genetic correlations

We used linkage disequilibrium score regression, version 1.0, to calculate genetic correlations as previously described. Pleiotropic genomic variants or correlated causal genomic loci can give rise to genetic correlations. To estimate these genetic correlations, the effect size of the first trait at the corresponding genomic polymorphism is multiplied by the effect size of the second trait. The product is regressed on the linkage disequilibrium score and the slope reflects the genetic correlation between both traits [30].

### Part 2

In the second part of the analyses, we associated polygenic scores for persistent thinness, obesity, other anthropometry-related traits, and psychiatric disorders with adolescent persistent thinness in an independent population-based sample, the ALSPAC cohort. This approach can overcome the limitation of the Riveros-McKay et al. persistent thinness GWAS, which excluded all participants with any psychiatric illness. Further, using this external sample enabled us to investigate whether persistent thinness in adulthood (as defined by the GWAS) has similar genetic aetiology as adolescent persistent thinness defined by us in ALSPAC.

### Sample from the Avon Longitudinal Study of Parents and Children (ALSPAC)

During the period April 1, 1991, until December 31, 1992, the ALSPAC cohort was started by inviting pregnant women in the former county of Avon, United Kingdom, to participate in the developmental population-based cohort [31, 32]. The cohort originally included 14,541 pregnancies of whom 13,988 children were alive at one year and the cohort was boosted with 913 children at the age of seven years. For the exclusion of disordered-eating symptoms, follow-up was conducted at age 14 (wave 14, *n* = 10,581), 16 (wave 16, *n* = 9702), and 18 years (wave 18, *n* = 9505) with these response rates: 6140 (58%) responding at wave 14, 5069 (52%) at wave 16, and 3228 (34%) at wave 18. Additionally, parent-reported questionnaires are available for 7025 adolescents at wave 14 and on 5656 at wave 16 to strengthen the validity of the probable eating disorder diagnoses [33] (for diagnostic criteria, see Supplementary Table S2, and for numbers, see Supplementary Table S3). Please note that the study website contains details of all the data that are available through a fully searchable data dictionary and variable search tool (www.bristol.ac.uk/alspac/researchers/our-data/). To assure genetic unrelatedness of participants in our analyses, we randomly selected one individual of each closely related pair (*φ* > 0.2) using PLINK v1.90 [34], excluding 75 (n_female_ = 37, 49.3%) participants.

### Genotyping, imputation and quality control in the Avon Longitudinal Study of Parents and Children (ALSPAC)

Genome-wide genotyping was performed on 9915 of 15,247 children participating in ALSPAC using the Illumina HumanHap550 quad chip. Participants with a SNP missingness > 3%, insufficient sample replication (identity by descent < 0.1), biological sex mismatch, and non-European ancestry (as defined by multi-dimensional scaling using the HapMap Phase II, release 22, reference populations) were excluded. All SNPs underwent the following quality control: They were excluded if their minor allele frequency (MAF) was < 1%, excessive missingness occurred (i.e., call rate < 95%), or a departure from the Hardy–Weinberg equilibrium had a *p*-value < 5 × 10^−7^. Genotypes were imputed with Impute3 to the Human Reference Consortium (HRC) 1.0 reference panel and phased using ShapeIT (v2.r644). After imputation, any SNPs with MAF < 1%, Impute3 information quality metric of < 0.8, and Hardy-Weinberg equilibrium *p* < 5 × 10^−7^ were excluded. After quality control, 8654 participants (4225 females and 4429 males) and 4,054,653 SNPs could be carried forward for analyses.

### Latent class growth analysis to identify individuals with adolescent persistent thinness

We identified participants with adolescent persistent thinness analysing objectively measured BMI (kg/m²) collected at 10, 12, 13, 14, 16, 18, and 24 years in ALSPAC [35]. These BMI measures were highly correlated (see Supplementary Fig. S1). Prior to analysis, BMIs at each time point were log-transformed and dichotomised in the bottom 5% versus remaining 95%. Participants who engaged in any weight loss behaviours (e.g., fasting, purging, and/or laxative use) at 14, 16, or 18 years, or met criteria for anorexia nervosa, bulimia nervosa, and/or purging disorder (*n* = 791) were excluded, for detailed descriptive statistics and number of excluded participants at each wave see Supplementary Table S3, 4. We derived longitudinal latent classes using latent class growth analysis with a full information maximum likelihood [36, 37] for all participants using Mplus version 8.4. For detailed description of this method and fit statistics, see Supplementary Table S5 and Supplementary Methods.

A model with three classes fit the data best. The majority (*n* = 7884, 93%) of participants were never in the class of the lowest 5% of logBMI. Participants assigned to the second class (*n* = 349, 4%) had a 0.25 probability of being in the lowest 5% of the logBMI distribution, whereas participants in class three (*n* = 272, 3%) were highly likely to be in the lowest 5% of the logBMI distribution at all time points (Supplementary Fig. 2). Class three was considered to represent participants with adolescent persistent thinness and classes one and two were collapsed creating the control group. The resulting two groups were carried forward in the following analyses: individuals with adolescent persistent thinness (*n* = 272; 3%) and individuals without thinness (*n* = 8233; 97%). For illustration, the mean BMI and standard deviation at each wave, for each class, are listed in Supplementary Table S6. Only a subsample of these participants had both phenotype and genotype data: 203 cases (*n*_female_ = 80, 39.4%) and 6391 controls (n_female_ = 3147, 49.2%).

### Polygenic risk scoring in ALSPAC

We calculated polygenic scores of psychiatric disorders and anthropometric traits with PRSice [38], version 2.2.3. We clumped SNPs to obtain genetically independent SNPs present both in the discovery GWAS and ALSPAC. We retained the SNP with the lowest *p* value in each 250 kilobase window of all those in linkage disequilibrium (*r*^*2*^ > 0.1). We calculated eleven polygenic scores for psychiatric disorders and 13 anthropometry-related polygenic scores, including BMI [39], overweight, obesity class 1–3, extreme BMI [40], hip and waist circumference [41], childhood obesity [42], age at menarche [43], and persistent thinness [11] at their optimal *p*-value threshold in each individual of our subsample (for a full list of discovery GWAS, see Supplementary Table S1). For this, we weighted the number of effect alleles by the corresponding allele effect size across the remaining SNPs. We used high-resolution scoring (i.e., incrementally across a large number of *p*-value thresholds) to obtain the *p*-value threshold at which the polygenic score is optimally associated with the outcome and explains the most variance (i.e., resulting in Nagelkerke’s *R*^*2*^ on the liability scale). We fitted logistic regressions with adolescent persistent thinness as the outcome, adjusted for sex and the first six principal components. We calculated empirical *p* values by permuting case-control status at every *p*-value threshold 10,000 times to account for potential overfitting. We converted the observed *R*^*2*^ to the liability scale using the sample prevalence of adolescent persistent thinness as population prevalence using the approach implemented in the PRSice software based on the method by Lee et al. [44].

We calculated *Q* values using the false discovery rate approach to correct for 24 polygenic score regression models. We did not stratify analyses by sex due to the few individuals with adolescent persistent thinness, but included sex as a covariate.

## Results

### Results of Part 1: Genetic correlations of psychiatric disorders with persistent thinness

**Fig. 1:**
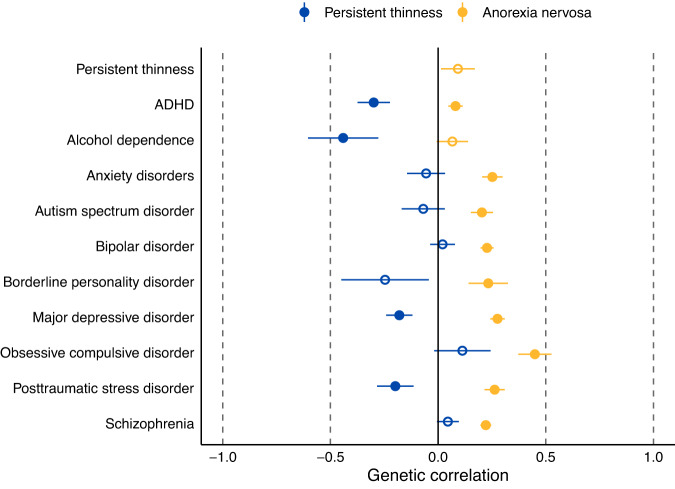
Results of Part 1: Genetic correlations of persistent thinness and anorexia nervosa. The plot shows genetic correlations of persistent thinness (blue) and anorexia nervosa (yellow) with psychiatric disorders calculated by linkage disequilibrium score regression. Filled dots are statistically significant after adjustment for multiple testing through the false discovery rate approach. Dots represent genetic correlations (r_g_) and error bars index standard errors. Genetic correlations were calculated using linkage disequilibrium score regression or anxiety disorders and obsessive-compulsive disorder were drawn from a previous publication (Watson et al. 2019) because no new GWAS had emerged since the publication of Watson et al. 2019. ADHD = attention deficit hyperactivity disorder.

The genetic correlation between the anorexia nervosa GWAS [13] and the persistent thinness GWAS [11] was modest and not statistically significant (*r*_g_ = 0.09, se = 0.08, *p* =  0.34). In contrast to anorexia nervosa, persistent thinness showed a statistically significant negative genetic correlation with major depressive disorder (r_g_ = −0.18, se = 0.06, Q = 0.01) and posttraumatic stress disorder (r_g_ = −0.20 se = 0.08, Q = 0.03), ADHD (*r*_g_ = −0.30, se = 0.08, *Q* = 1.79 × 10^−4^) and a stronger negative correlation with alcohol dependence (*r*_g_ = −0.44, se = 0.16, *Q* = 0.01, Fig. 1). Anorexia nervosa, in contrast, showed positive genetic correlations with lifetime anxiety disorders (*r*_g_ = 0.25, se = 0.05, *Q* = 8.90 × 10^−8^), autism spectrum disorder (*r*_g_ = 0.20, se = 0.05, *Q* = 1.79 × 10^−4^), bipolar disorder (*r*_g_ = 0.23, se = 0.03, *Q* = 3.94 × 10^−^^13^), borderline personality disorder (*r*_g_ = 0.23, se = 0.09, *Q* = 0.02), major depressive disorder (*r*_g_ = 0.28, se = 0.03, *Q* = 1.93 × 10^−^^15^), obsessive-compulsive disorder (*r*_g_ = 0.45, se = 0.08, *Q* = 2.61 × 10^−8^), posttraumatic stress disorder (*r*_g_ = 0.26, se = 0.05, *Q* = 8.2 × 10^−8^) and schizophrenia (*r*_g_ = 0.22, se = 0.02, *Q* = 5.77 × 10^−19^). In summary, persistent thinness shows negative genetic correlations with psychiatric disorders whereas anorexia nervosa shows positive genetic correlations. For all calculated genetic correlations, see Supplementary Table S7.

### Results of Part 2: Polygenic scores associated with adolescent persistent thinness

The effect sizes are expressed as odds ratios (OR) per standard deviation increase of polygenic score (for a full list of results, see Supplementary Table S8). At the current sample size of the persistent thinness GWAS, the persistent thinness polygenic score (OR = 1.16, 95% CI: 1.00, 1.33; *Q* = 0.56) was not associated with adolescent persistent thinness in ALSPAC.

**Fig. 2:**
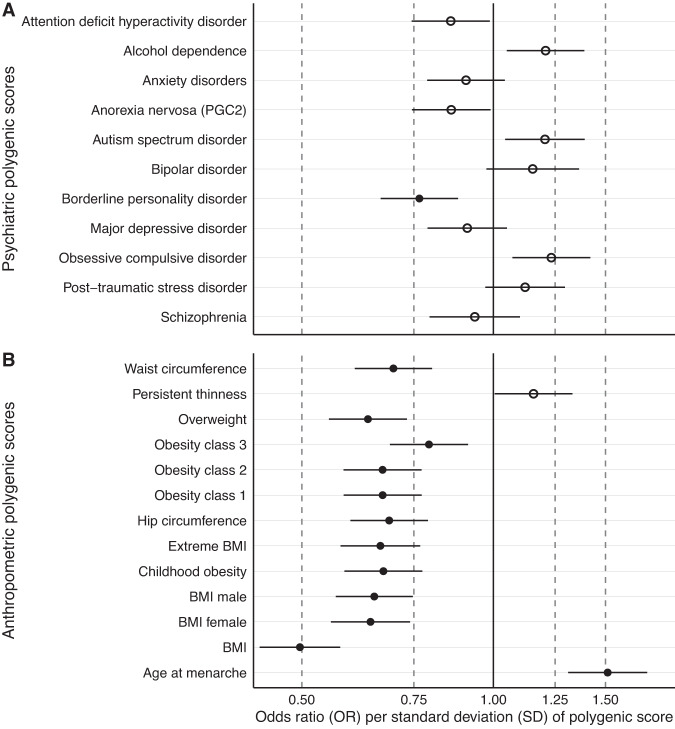
Results of Part 2: Panel A & B Polygenic scores associated with adolescent persistent thinness in the Avon Longitudinal Study of Parents and Children (ALSPAC). **A** Shows psychiatric and (**B**) anthropometric polygenic scores and their association estimates with latent class growth analysis-derived adolescent persistent thinness (*n* = 8505). Filled dots are statistically significant after adjustment for multiple testing through the false discovery rate approach. Dots represent odds ratios (ORs) and error bars index 95% confidence intervals obtained via logistic regression and 10,000 permutations to obtain empirical *p* values. BMI = body mass index, PGC2 = second freeze of the anorexia nervosa genome-wide association study of the Psychiatric Genomics Consortium.

An increased genetic predisposition for borderline personality disorder (OR = 0.77, 95% CI: 0.67; 0.88; *Q* = 0.01) was associated with lower risk of adolescent persistent thinness (Fig. 2A).

Nine anthropometric polygenic scores were negatively associated with adolescent persistent thinness (i.e., age 10–24 years; Fig. 2B and see Supplementary Table S8 for full results). A genetic predisposition to a higher BMI (OR = 0.50, 95% CI: 0.43, 0.57; *Q* = 2.40 × 10^−4^) was associated with a lower risk of adolescent persistent thinness. Overall, the associations with anthropometric polygenic scores were stronger than with psychiatric polygenic scores. Consistently, a higher genetic predisposition to childhood obesity (OR = 0.67, 95% CI: 0.58; 0.77; *Q* = 2.4 × 10^−4^) was associated with a lower risk of adolescent persistent thinness. The childhood (OR = 0.67) and adulthood (OR = 0.67) obesity polygenic scores showed similar effect sizes of their association with adolescent persistent thinness with overlapping confidence intervals (CIs). Similarly, male- or female-specific BMI polygenic scores showed no differences in the effect sizes of the associations (OR_men_ = 0.65; OR_women_ = 0.64 with overlapping CI). In contrast to anthropometric polygenic scores, the age at menarche polygenic score was positively associated with adolescent persistent thinness (OR = 1.51, 95% CI: 1.31, 1.74; *Q* = 2.4 × 10^−4^).

## Discussion

In Part 1 of our study and in line with our hypotheses and previous research [11], we demonstrated that persistent thinness shows no significant genetic correlation with anorexia nervosa, indicating that they may be genomically distinct. To better understand differences in their genetic architecture, we calculated genetic correlations with other psychiatric traits.

### Psychopathology

In line with our hypothesis, in Part 1 of our study, four of eleven investigated psychiatric disorders (ADHD, alcohol dependence, major depressive disorder, and post-traumatic stress disorder) showed a negative genetic correlation with persistent thinness, but not with anorexia nervosa. This means that these phenotypes share genetic variants with persistent thinness but with opposite directions of effect. In Part 2 of our study, we showed that individuals with adolescent persistent thinness carried lower polygenic scores for borderline personality disorder, suggesting further that persistent thinness was not associated with increased genetic risk for psychiatric disorders. Phenotypically, individuals with thinness demonstrate less psychopathology or no disordered-eating behaviours [3], supporting our genetic findings.

Overall, in contrast to thinness, anorexia nervosa showed strong positive genetic correlations with obsessive-compulsive disorder, schizophrenia, anxiety disorders, and bipolar disorder, whereas persistent thinness showed no genetic correlations with these disorders (Fig. 1). This indicates clearly that whereas anorexia nervosa shares genetics with several other psychiatric disorders, persistent thinness does not.

### Anthropometry

**Fig. 3:**
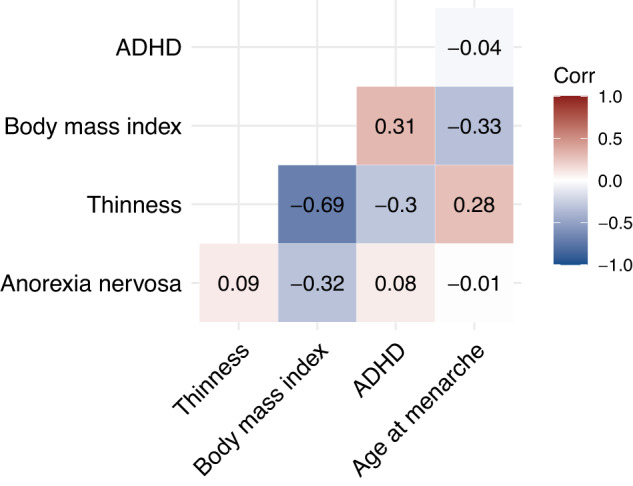
Genetic correlation heatmap. Genetic correlations among persistent thinness, anorexia nervosa, attention deficit hyperactivity disorder (ADHD), body mass index, and age at menarche. Genetic correlations were calculated using linkage disequilibrium score regression.

In Part 2 of our study, as hypothesised, individuals with adolescent persistent thinness carried lower BMI, hip, and waist circumference polygenic scores than individuals without thinness, replicating a finding from the original GWAS that reported a negative genetic correlation between thinness and BMI (*r*_g_ = −0.69; Fig. 3) [11]. Furthermore, in the ALSPAC cohort, we showed that individuals with adolescent persistent thinness carry higher age at menarche polygenic scores than individuals with higher BMIs, suggesting that late menarche and thinness share genetics. This finding is expected, because BMI and age at menarche are negatively genetically correlated (*r*_g_ = −0.33; Fig. 3) [45, 46]. These genetic correlations indicate that body weight regulation and pubertal timing have shared genetic underpinnings that are also relevant for extreme weight phenotypes like persistent thinness.

However, adding anorexia nervosa to the picture suggests more complicated mechanisms: like thinness, anorexia nervosa shows a negative genetic correlation with BMI [13, 47]. However, thinness and anorexia nervosa are not genetically correlated with each other [11]. This suggests that two different sets of genetic variants may be operative: one set shared between thinness and BMI, and one set shared between anorexia nervosa and BMI.

According to this threeway relationship, anorexia nervosa shows no genetic correlation with ADHD [13, 14, 48]. However, both ADHD and thinness are genetically correlated with each other and with BMI [47, 49]. Again, this suggests two sets of genetic variants may exist: one set of genetic variants shared among thinness, BMI, and potentially ADHD that may be distinct from the set shared genetics between anorexia nervosa and BMI.

### Persistent thinness phenotypes

In Part 2 of our analysis, the persistent thinness polygenic score and our latent class growth analysis-derived adolescent persistent thinness phenotype in the ALSPAC cohort showed an expected positive association; however, the association was not statistically significant at our multiple testing threshold. We offer three potential explanations. First, the age mismatch between the discovery GWAS (i.e., 18–64 years) and our adolescent persistent thinness phenotype (i.e., 10–24 years) may have contributed to this finding, because GWASs of BMI are not perfectly correlated across age [50], suggesting that different genetic variants influence BMI at different ages. Second, we may have had limited statistical power to detect an association as both ALSPAC (*n* = 272) and the discovery GWAS (*n* = 1471) included relatively few individuals with thinness. Third, our adolescent persistent thinness phenotype in ALSPAC may not have been defined strictly enough, because we were unable to exclude participants with somatic or psychiatric illnesses that could lead to underweight. Increasing the sample sizes of both the target samples as well as the discovery GWAS may clarify our preliminary finding.

Further limitations regarding our adolescent persistent thinness phenotype must be acknowledged. Some participants may develop obesity after age 24 years [12, 51] when our longitudinal modelling ended. Longitudinal studies suffer from attrition; however, in our study, over 70% had at least three or more measures of BMI. Further, as shown in Supplementary Figure S^1^, BMI from age 10 to 24 in the ALSPAC sample is highly correlated which supports our longitudinal modelling approach that identified participants with adolescent persistent thinness.

In Part 1 of our study measuring genetic correlations, the definition of thinness in the published GWAS excluded individuals with psychiatric disorders, potentially accounting for the lack of genetic correlations between thinness and psychiatric disorders. Importantly, in Part 2 of our study, where we intentionally did not exclude individuals with psychiatric disorders, taking medication, with weight regulation-affecting physical conditions, using illicit substances, or who smoked [12], we nonetheless observed a pattern of associations with polygenic scores similar to that observed when those with psychiatric disorders were excluded (Fig. 2A vs Fig. 1).

In addition, by nature of the available data, we only investigated genomics in white British participants. It is essential that future studies include larger and more diverse populations to ensure that our genetic investigations accurately capture the ways in which genes influence disease risk in ancestrally diverse populations [52].

In Part 2, in contrast to previous papers, our analysis benefitted from repeated measurements across development that afforded a more precise phenotype than cross-sectional thinness measures. In this context, it is important to highlight that longitudinal studies, such as ALSPAC, suffer from attrition, whereby a non-random selection of families continues to participate. This can lead to selection bias and challenge the representativeness of the sample. In the context of our study, unmeasured confounders may be associated with the decision to provide a saliva sample for DNA extraction and continuing participation in subsequent waves. This unmeasured confounding could bias results, therefore, it is important for future research to replicate and triangulate our findings in different populations [53]. Additionally, excluding individuals with disordered eating behaviour and eating disorders from our young people with thinness reduced the risk of identifying false positives.

To unravel the complex relationships among ADHD, thinness, anorexia nervosa, body mass index, and pubertal timing, future investigations should model these genetic influences concurrently. In addition, researchers should apply longitudinal modelling approaches to chart trajectories of growth and symptoms across development. Together joint and longitudinal modelling may answer the question of which trait or traits are responsible for the shared genetics between psychiatric disorders and thinness. For example, genomic structural equation modelling would be a useful approach. Moreover, transdiagnostic GWAS of genetically associated traits or disorders may clarify shared genetic factors. In summary, our findings suggest that thinness and anorexia nervosa may differ on a genomic level that may be characterised by differential genomic liability for psychiatric illness and body weight regulation.

### Supplementary information

Supplementary Methods & Figures S1 & S2

Supplementary Tables S1-S8

## Data Availability

Genetic correlations: All data analysed during this study are included in its supplementary information files as link to the original downloadable GWAS summary statistics. Polygenic score analyses: The data that supports the findings of this study are available from the University of Bristol but restrictions apply to the availability of these data, which were used under license for the current study, and so are not publicly available. Data are however available from the authors upon reasonable request and with permission of the University of Bristol. Researchers can apply for data access on the following website: http://www.bristol.ac.uk/alspac/.
