# Supplementary material for: Persistent thinness and anorexia nervosa differ on a genomic level
Source: Eur J Hum Genet. 2023 Jul 20;32(1):117–24. doi: 10.1038/s41431-023-01431-8 (PMC10772076; doi:10.1038/s41431-023-01431-8)
Supplement: Supplementary file 1 — Supplementary Methods & Figures S1 & S2 [file 41431_2023_1431_MOESM1_ESM.docx]

**Persistent thinness and anorexia nervosa differ on a genomic level**

Christopher Hübel^1,2,3,4,5^*, Mohamed Abdulkadir^3,6^*, Moritz Herle^1,7^*, Alish B. Palmos^1,2^, Ruth J. F. Loos^8,9^, Gerome Breen^1,2^, Nadia Micali^5,10,11⤉^ & Cynthia M. Bulik^4,12,13⤉^

^1^Social, Genetic & Developmental Psychiatry Centre, Institute of Psychiatry, Psychology & Neuroscience, King’s College London, UK

^2^National Institute for Health Research (NIHR) Maudsley Biomedical Research Centre at South London and Maudsley NHS Foundation Trust, London, UK

^3^National Centre for Register-based Research, Aarhus Business and Social Sciences, Aarhus University, Aarhus, Denmark

^4^Department of Medical Epidemiology and Biostatistics, Karolinska Institutet, Stockholm, Sweden

^5^Department of Pediatric Neurology, Charité – Universitätsmedizin Berlin, Berlin, Germany

^6^Department of Psychiatry, Faculty of Medicine, University of Geneva, Geneva, Switzerland

^7^Department of Biostatistics & Health Informatics, Institute of Psychiatry, Psychology & Neuroscience, King’s College London, UK

^8^Charles Bronfman Institute for Personalized Medicine, Icahn School of Medicine at Mount Sinai, New York, New York, USA

^9^ Novo Nordisk Foundation Center for Basic Metabolic Research, Faculty of Health and Medical Science, University of Copenhagen, Copenhagen, Denmark

^10^ Great Ormond Street Institute of Child Health, University College London, London, UK

^11^Mental Health Services in the Capital Region of Denmark, Eating Disorders Research Unit, Psychiatric Centre Ballerup, Ballerup, Denmark

^12^Department of Psychiatry, University of North Carolina at Chapel Hill, Chapel Hill, NC, USA

^13^Department of Nutrition, University of North Carolina at Chapel Hill, Chapel Hill, NC, USA

*contributed equally to this work

^⤉^shared senior authorship

**Supplementary Methods. Latent class growth analysis**

**Supplementary Figure S1. Correlations between body mass index measurements**

**Supplementary Figure S2. Latent class growth analysis results**

**Supplementary Methods**

In latent class growth analysis, the number of classes is not directly estimated and alternative specifications with increasing numbers of classes were compared. The best-fitting model was identified using the Akaike Information Criterion (AIC), Bayesian Information Criteria (BIC), and entropy. For AIC and BIC, lower values indicate better model fit, whereas a high entropy is desirable. Further, trajectory size is taken into consideration, as small (<3%) trajectories are hard to interpret. After selection of the optimal number of classes, estimations were repeated using 1000 random starts to avoid local maxima. A model with three classes was the best fit in comparison with the one, two, or four class solution (for a full list of fit statistics, see **Supplementary** **Table S5**).


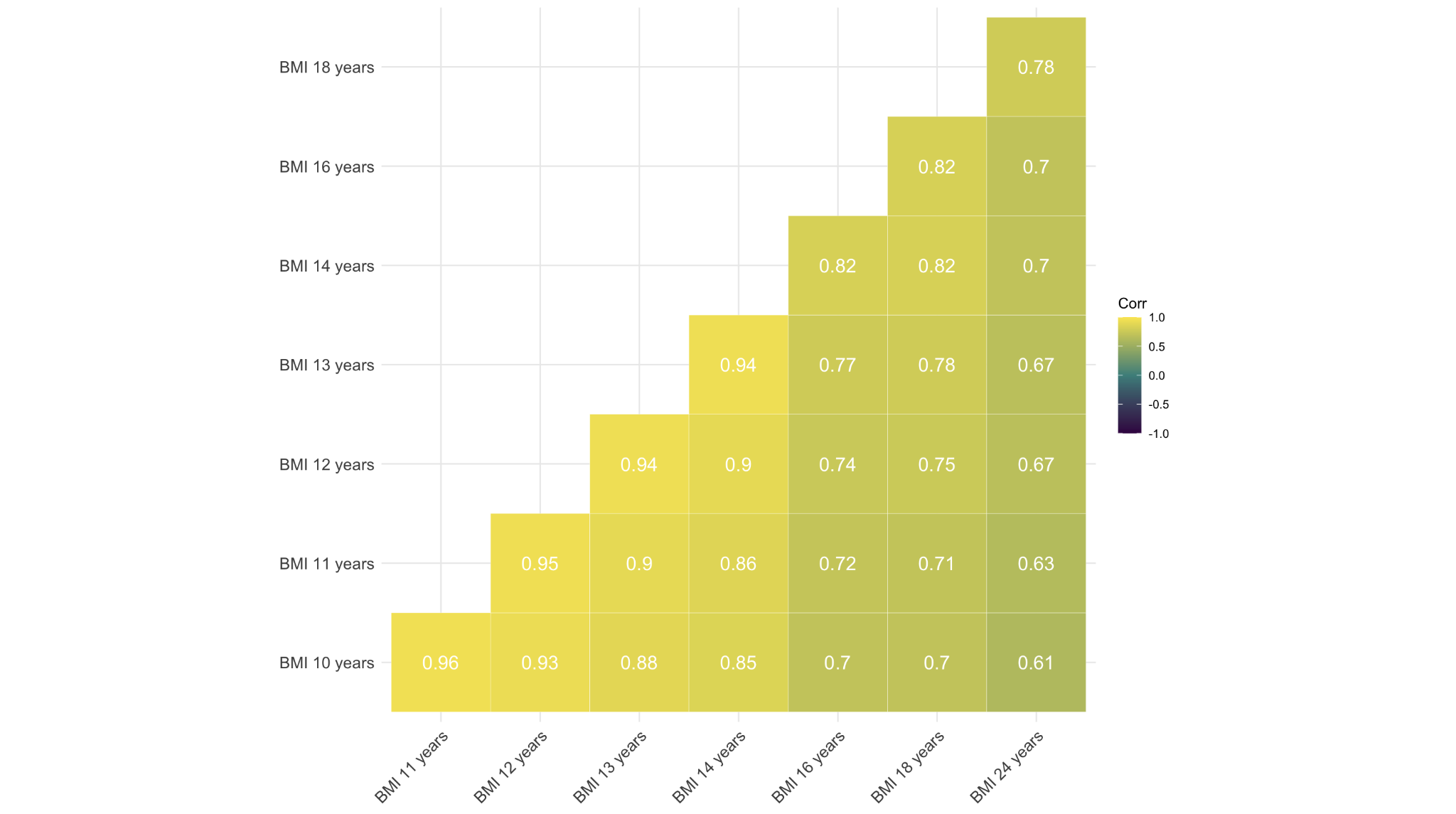


**Supplementary Figure S1.** Correlation plot of body mass index (BMI) from year 10 until 18 years. BMI is generally higher than 0.61 correlated across the different ages.


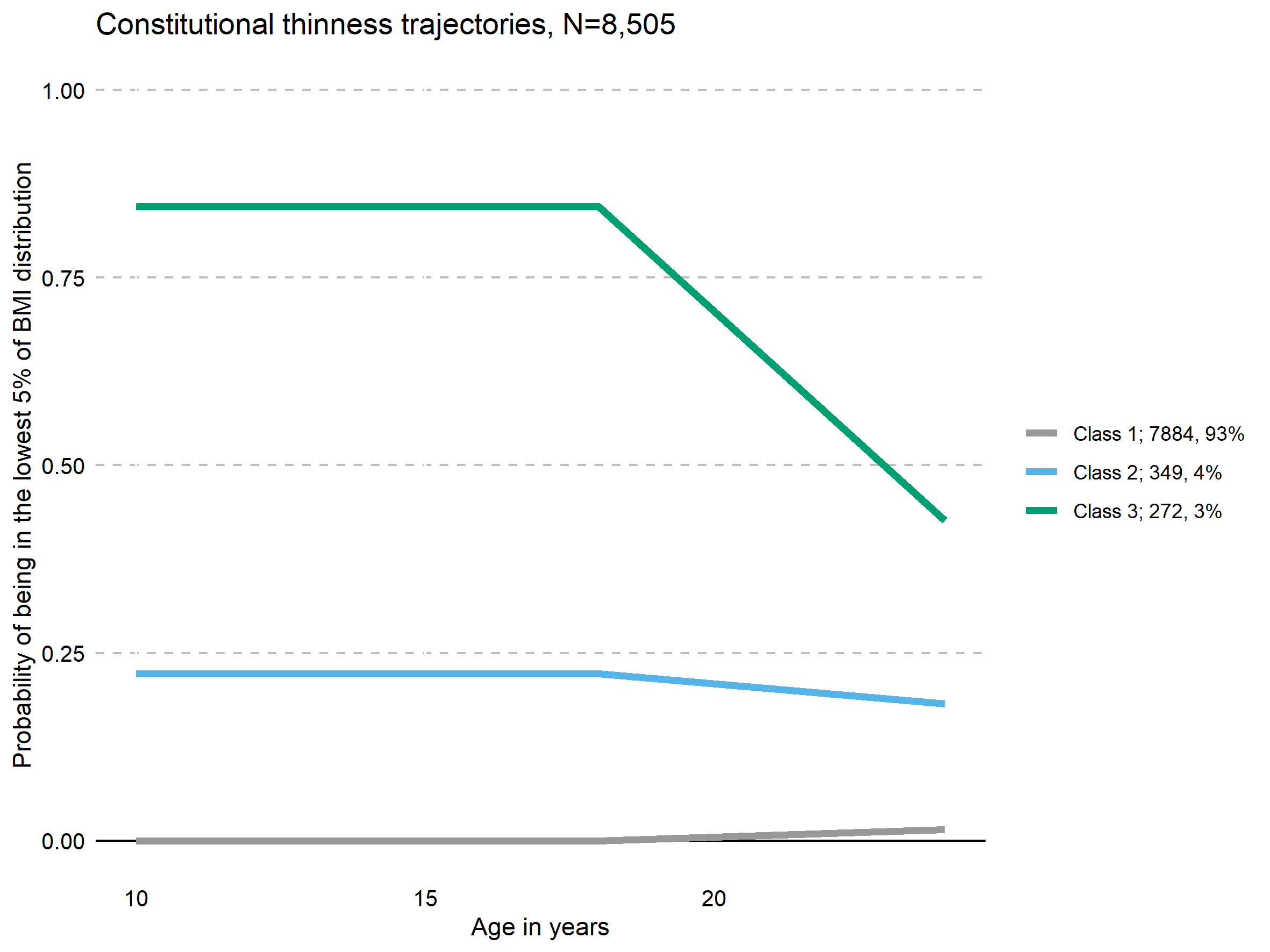
**Supplementary Figure S2.** Results from the latent class growth analyses of adolescent persistent thinness in the Avon Longitudinal Study of Parents and Children (ALSPAC, n = 8,505). Classes 1 and 2 were collapsed in our analysis as one comparison group (n = 8,156). We defined class 3 (green) as individuals with persistent thinness across adolescence and young adulthood in ALSPAC (n = 272).
